# Antimicrobial Effect of *Simira ecuadorensis* Extracts and Their Impact on Improving Shelf Life in Chicken and Fish Products

**DOI:** 10.3390/foods11152352

**Published:** 2022-08-05

**Authors:** Jorge F. Reyes, Ana M. Diez, Beatriz Melero, Jordi Rovira, Isabel Jaime

**Affiliations:** 1 Departamento de Química, Universidad Técnica Particular de Loja, San Cayetano Alto s/n, Loja 1101608, Ecuador; 2 Department of Biotechnology and Food Science, University of Burgos, 09001 Burgos, Spain

**Keywords:** *Simira ecuadorensis* extract, antimicrobial activity, pathogenic bacteria, spoilage bacteria, chicken broth, fish hamburgers

## Abstract

The objective of this work was to evaluate the antimicrobial potential of different extracts of *Simira ecuadorensis*, a characteristic plant of Ecuador, and to validate its potential as a food preservative. Four extracts referred to as ethanol, ethanol-water (50:50 *v/v*), spray-dried, and freeze-dried were obtained under different processes. Initially, their antimicrobial activities were evaluated against a wide group of microorganisms consisting of 20 pathogenic and spoilage microbial strains found in foods through the agar diffusion method. Then, the extracts with the best yields and antimicrobial properties against microorganisms of greatest interest were selected to determine their effect on model foods preserved under normal commercial conditions through challenge tests. Spray-dried and ethanol-water extracts were tested for their ability to inhibit *C. jejuni* in chicken model products, where is a common pathogen and *Shew. putrefaciens* in fish model products as it is a spoilage microorganism frequently found in fish. One solid and one liquid were chosen as model foods: burger and broth, respectively. *Campylobacter jejuni* and *Shewanella putrefaciens* were effectively inhibited by the four extracts with minimum inhibitory concentration (MIC) of 80 mg/mL. *Bacillus cereus*, *Yersinia enterocolitica*, *Clostridium perfringens*, and *Leuconostoc mesenteroides* were also inhibited by ethanolic extract. The ethanol-water extract showed greater antimicrobial activity in fish products, whereas spray-dried extract had low growth inhibition of *C. jejuni* in chicken burgers; however, it was quite effective on *C. jejuni* in broth. The spray-dried extract significantly decreased the pH of the chicken burgers, while the ethanolic extract had a slight impact on the pH of the fish burgers. The presence of antibacterial effects revealed that the *S. ecuadorensis* extracts could be potentially used in food preservation and as a natural antimicrobial.

## 1. Introduction

Ecuador is considered one of the countries possessing the highest biodiversity in the world [1]. The majority of the world’s population in developing countries use herbal medicine, and Ecuador is not an exception [2]. The use of medicinal plants is a traditional practice in Ecuador, and it is important to provide information about the species that could be used in the future for their properties in the food industry [3].

One of the most widely used chemical additives in the food industry are preservatives, which are used to maintain the food quality, improve its microbiological and chemical stability, extend shelf life, and prevent economic losses [4,5]. However, at present, the interest of both consumers and food manufacturers in the development of food products free of synthetic chemical additives has grown due to the various adverse effects that these have on human health (allergies, headaches, etc.). This has caused “green foods” to be considered of great interest, foods with additives of natural origin [6,7,8,9]. Bondi et al. and Cabral et al. [7,10] present several investigations that attribute antimicrobial and antioxidant activities to plant extracts, essential oils, and functional ingredients, such as phenols, polyphenols, flavonoids, alkaloids, and tannins, which provide certain physiological action on cells [11]. In the scientific literature, it is mentioned that there are at least three mechanisms of antimicrobial action of phenolic compounds: (i) modification of the permeability of cell membranes, formation of cytoplasmic granules, and rupture of the cytoplasmic membrane; (ii) changes in various intracellular functions induced by hydrogen bonding of the phenolic compounds with enzymes through their OH groups; and (iii) modification of fungal morphology as a result of different interactions with cell membranes [8,10].

*Simira ecuadorensis* (Standl.) Steyerm is a small tree characteristic of the Ecuadorian dry forest [12], grows up to 10 m tall, is branched and sometimes has many stems, and sprouts from December [13]. Its wood is used for rural constructions, its branches and stems are used for orchard fences and firewood, and its leaves are used to wrap goat cheeses to preserve them and give them flavor/color [14]. Rondón et al. [15] quantified the total phenolic content and evaluated the antibacterial capacity of 13 native species collected in the province of Guayas-Ecuador, including *S. ecuadorensis*. In the tests against three different microorganisms, the ethanolic extracts were the ones that presented the highest antibacterial activity, which could be attributed to the presence of phenolic compounds (phenolic acids, flavonoids, tannins, coumarins, quinones). They were mainly effective against *Vibrio parahaemolyticus* at moderate concentrations. In addition, these authors recommend studying the effect of the extracts against a greater number of bacterial species. The scientific literature related to the composition and application of this plant is rather scarce. Therefore, the aim of this research is the study of the effect of *S. ecuadorensis* extracts on bacterial growth “in vitro” and its application in real food systems.

## 2. Materials and Methods

### 2.1. Materials

#### 2.1.1. Plant Material

Fresh leaves of *S. ecuadorensis* (Standl.) Steyerm, “guápala” were collected in December 2019 from Zapotillo in Loja province, Ecuador. These were cleaned and dried using a tray dryer (DY-110H, Daeyeong E&B CO., Ltd., Ansan, Korea) at 40 °C for about 12 h. Samples were ground, sifted, and homogenized (particle size range < 350 µm) before extraction and were stored at refrigerated conditions until the process was carried out.

#### 2.1.2. Extracts

The dried and ground leaves were extracted with water, ethanol, and ethanol-water (50:50 *v/v*) as solvents; five liters of solvent were used for each kilogram of leaves. It was left to rest in amber containers for three days at refrigeration temperature, stirring if necessary. In the case of the two ethanolic extracts, the ethanol was evaporated in a rotary evaporator (G1, Heidolph, Schwabach, Germany) at 40 °C and 150 rpm, and the water was removed by lyophilization in a freeze-drier (Labconco, Kansas City, MO, USA) with the following settings: pressure = 0.180 mbar, temperature = −50 °C for 72 h; these extracts are henceforth designated as ETOH and ETOH-H_2_O. As for aqueous extracts, one part was frozen at −40 °C and freeze-dried (as described above) and the other part was mixed with maltodextrin (3%) and spray-dried (Büchi Mini Spray Dryer B-290, Switzerland) at 120 °C and 6 mL/min of feed flow rate; they were designated as H_2_O and ATOM, respectively. The yields of ETOH, ETOH-H_2_O, H_2_O, and ATOM extracts were 1.04%, 3.99%, 6.98%, and 12.41%, respectively.

### 2.2. Experimental Scheme

Initially, since information on the antimicrobial activity of *S. ecuadorensis* is scarce, a first in vitro study was planned to generate a database, testing the effect of the extracts against 20 species of pathogenic and spoilage microorganisms found in foods.

In a second step, a study of the effect of the extracts on the relevant species selected in the “in vitro” phase (*C. jejuni* and *Shew. putrefaciens*) inoculated into model foods was carried out. As model foods to perform the challenge tests, those in which these microorganisms are usually found (chicken and fish products, respectively) were chosen.

### 2.3. Antimicrobial Activity “In Vitro”

#### 2.3.1. Microbial Species

The strains were obtained from the Spanish Collection of Type Cultures (CECT), the International Institute of Life Sciences (ILSI) and the University Hospital of Burgos-University of Burgos (HUBU-UBU) and they are collected in Table 1.

#### 2.3.2. Determination of Antimicrobial Activity

Antimicrobial activity of all plant extracts was assessed using a modified agar diffusion method [4]. The test bacteria were grown until they reached a count of approximately 10^8^ cfu/mL. Bacterial suspension was spread on the solid medium plates. After solidification of the medium, 7 mm-diameter wells were made in plates using a sterilized pipette. The extracts were diluted to 80 mg/mL, using deionized water and dimethyl sulfoxide (DMSO) (1:1), and introduced into the wells (40 μL). The plates were incubated for 24 to 48 h and the antimicrobial activity of samples were determined by measuring the diameter of the colony-free perimeter using a caliper. Water-DMSO solution was used as negative control, while a sodium hypochlorite solution (0.94%) was used as positive control. All the tests were performed in triplicate. Culture conditions of the selected microorganisms are presented in Table 1.

The minimum inhibitory concentration (MIC) was determined only with microorganisms that displayed inhibitory zones. *S. ecuadorensis* extracts were diluted in a water-DMSO solution and the following concentrations were tested: 40, 20, 10, and 5 mg extract/mL, and the agar diffusion method described above was carried out. The lowest concentration (mg/mL) of extract that visibly inhibited the growth of the tested bacteria was considered as the MIC value. The assays were performed in triplicate.

### 2.4. Challenge Test

Chicken and fish commercial broths (Aneto, Spain) and frozen chicken and hake fillets (Carrefour, Spain) were used to evaluated the effect of extracts on foods.

#### 2.4.1. Inoculum Preparation

The two reference *C*. *jejuni* strains CECT 7572 and ATCC 11118, together with the strain HUBU-UBU 410 isolated from a clinical case at the Burgos hospital, were grown under the same conditions previously described (Table 1). Plates were incubated at 42 °C for 48 h under microaerobic atmosphere (5% O_2_, 10% CO_2_, 85% N_2_) generated by CampyGen^®^ from Oxoid LTD (Hampshire, UK). One colony from Nutrient 5% Blood Agar from Oxoid LTD (Hampshire, UK) was transferred into BHI Broth from Oxoid LTD (Hampshire, UK) and was incubated as described above. The cultures were then centrifuged at 7000 rpm for 10 min at 4 °C. The supernatant was decanted, and the pellet was suspended in 10 mL sterile water by vortexing to achieve a viable cell population of 6 log cfu/mL. Then, the 3 tubes containing 10 mL of the cell suspension for each strain were mixed and 1% of this suspension was mixed with chicken meat to obtain a final concentration of 4 log cfu/g and 0.1% with chicken broth to obtain 3 log cfu/mL.

*Shew. putrefaciens* (CECT 5346) and *Shewanella* sp (CECT 4640) strains were grown at 37 °C overnight in TSB broth from Oxoid LTD (Hampshire, UK), and the concentration was adjusted with sterile water by optical density to 8 log cfu/mL. Both suspensions were mixed and diluted to obtain a viable cell population of 6 log cfu/mL. As in the previous product, 1% of the inoculum was added to minced fish and 0.1% to the broth.

#### 2.4.2. Chicken and Fish Products

The fillets were safely thawed under refrigeration and ground with a blender (A320, Moulinex) for 2 min in order to obtain a paste which was split into four fractions, one for each treatment. Small chicken/fish burgers (20 g each) were prepared, and three of each of them were placed in a polyethylene/ethylene vinyl alcohol/polystyrene tray and packed in a modified atmosphere (MAP; 30% CO_2_, 70% N_2_ and 60% CO_2_, 40% N_2_ for chicken and fish, respectively) using a WITT-Gasetechnik mixer and a packaging machine (Efabind, Murcia, Spain). Commercial sterilized broth “Aneto” (chicken and fish) was used; 1 mL was dosed in sterile Eppendorf’s. All samples were stored at 4 °C.

Four batches of chicken meat burgers and broth samples were prepared and codified as follows: C, control (without extracts), E with extract ATOM (8%), J with inoculum (*C. jejuni*), and EJ with inoculum and extract. The meat for J and EJ samples were mixed with 1% of *C. jejuni* inoculum previously described. In burgers, the final concentration of *C. jejuni* was 4 log cfu/g and the samples were analyzed at 0, 1, 4, 7, and 10 days, while in broth samples, the concentration of *C. jejuni* was 3 log cfu/mL, and they were analyzed after 0, 1, 4, 7, 10, 14, 29, 52, 67, and 85 days.

In the case of fish, the preparation of the samples was similar: C, control (without extract), E with extract ETOH-H_2_O (8%), S with inoculum, and SE with inoculum and extract. The samples S and SE were prepared with *Shewanella* inoculum in the same way that the chicken samples were prepared. The final concentration of *Shewanella* was 4 log cfu/g in burgers, and the samples were analyzed at 0, 1, 3, 7, 9, and 14 days. The final concentration in broth was 3 log cfu/mL, and the samples were analyzed at 0, 1, 3, 7, 14, 21, 28, 38, 44, 65, 72, 84, 104, and 131 days. All batches were stored at 4 °C.

#### 2.4.3. Microbiological Analysis

20 g of burgers were aseptically transferred to a sterile blender bag with a full-surface filter (BagPage, Brussel, Belgium) and homogenized with 180 mL of Ringer Solution (Oxoid, Barcelona-Spain) using a stomacher (Stomacher 400, Seward, London, UK) for 2 min. 100 μL of broth samples were taken directly from the Eppendorf and for both types of products, decimal dilutions were prepared as necessary and a superficial spreading was carried out in the corresponding media in order to perform the following determinations: Chicken burgers and broth: Aerobic mesophilic bacteria (AMB) on pour plates of Plate Count Agar (PCA) from Conda (Madrid, Spain) incubated for 24 h at 30 °C; *C. jejuni*, on spread plates of *Campylobacter* blood-free selective agar (CCD) from Oxoid LTD (Hampshire, England) and incubated at 42 °C for 48 h, under microaerobic condition.

Fish burgers and broth: Aerobic mesophilic bacteria (AMB) were determined on pour plates of Trypticase soy agar (TSA) from Oxoid LTD (Hampshire, England); and *Shew. putrefaciens*, on spread plates of iron agar from Conda (Madrid, Spain), both incubated for 24 h at 30 °C.

#### 2.4.4. pH Determination

pH was measured with pH-meter (micropH2001, CRISON, Barcelona, Spain) by placing electrode into the sample and using phosphate buffer solutions (pH 4.0 and 7.0) for calibration. Three measurements were done by changing electrode insertion place.

### 2.5. Statistical Analysis

Results are expressed as a means ± standard deviations. The differences among means were determined by one-way ANOVA using statistical package Statgraphics XVII-X64. Tukey’s test was used to determine if there are significant differences among the treatments at *p* < 0.05.

## 3. Results and Discussion

### 3.1. Antimicrobial Activity

Antimicrobial activity of four *S. ecuadorensis* Standl. extracts were screened against twenty bacteria, and obtained results are summarized in Table 2. Seven bacteria were affected, and an inhibition halo was produced that ranged from 12 to 18 mm, showing MIC values from 20 to 80 mg/mL (Table 3).

In this research, ETOH extract was shown to have the greatest antimicrobial effect by inhibiting the growth of *L. mesenteroides, Shewanella* sp., *Y. enterocolitica*, *C. perfringens*, and *B. cereus*, which may be due to the presence of phenolic compounds, such as anthraquinones that are extracted with ethanol, since alcoholic solvents are better to achieve higher level of phenolic compounds [16,17].

MIC values were 80 mg/mL for all the extracts; this value is high compared to that reported by Rondón et al. [15] for ethanolic extract of *S. ecuadorensis* showed antimicrobial activity against *V. parahaemolyticus* with MIC values of 40 ppm. Elez Garofulić et al. [18] and Sterniša et al. [19] reported high antimicrobial activity against strains of *C. jejuni* (MIC less than 0.512 mg/mL) and *Shew. putrefaciens* (2 and 3.13 mg/mL) using extracts of *Urtica dioica* L.

Moreira et al. [20] and Rondón et al. [15] reported that *S. ecuadorensis* has antimicrobial activity, and this is linked to its secondary metabolites; the most abundant are alkaloids whose mechanisms of action can affect cell division, cause respiratory inhibition and enzymatic inhibition in bacteria, alteration of the bacterial membrane, and involvement of virulence genes [21]. On the other hand, there are phenolic compounds whose mode of action is based on iron deprivation or hydrogen bonding with vital proteins and/or the loss of function of adhesins, cell wall polypeptides, and membrane-bound enzymes [22].

*S. ecuadorensis* has been little studied. There have been no reports of its use as an antimicrobial additive in food or the direct application of its extracts in food matrices, which highlights the importance of this study.

To develop the second part of the study on model foods, it was intended to test an ethanolic and an aqueous extract, since, as described above, the type of solvent influences the extract composition. As explained above, the antimicrobial activity was higher in ETOH, but its low extraction yield made further studies with it unfeasible. Then, it was not considered for application in food matrices, choosing ETOH-H_2_O as ethanolic extract. Among the two aqueous extracts having similar activity, the one with the highest yield was also selected (ATOM).

### 3.2. Effect of Spray-Dried Extract on Microbial Growth in Chicken Burgers during Storage

*Campylobacter* is a Gram-negative, thermophilic obligate microaerophilic bacterium that persists along the poultry food chain [23], usually by contamination of the carcass with intestinal contents during the slaughter process [24,25]. In many countries, *C. jejuni* is the agent responsible for more than 90% of cases of campylobacteriosis. Symptoms range from mild gastroenteritis to dysentery, although nongastrointestinal sequalae such as reactive arthritis, Guillain–Barré, and Miller–Fisher syndromes may appear [26,27]. Assessment of the antimicrobial potential of *S. ecuadorensis* extracts continued, measuring their inhibitory effect on the growth of AMB and *C. jejuni* in two chicken products (burgers and broth), chosen with consideration of what was mentioned in the previous paragraph. High initial values (>4 log cfu/g of bacteria) of AMB (day 0) in the control sample could be due to natural chicken meat contamination. Tamkutė et al. [28] indicated that the scientific community established that meat products are not suitable for consumption when AMB reaches 7–8 log cfu/g. C and J samples reached 8 log ufc/mL after 4 days of storage, while samples with extract (E and EJ) did not exceed these counts until day 7. At the end of storage, the counts reached approximately 8.8 log cfu/g for all treatments (Table 4). In the inoculated chicken burgers (EJ), the growth of *C. jejuni* was not significantly inhibited by the addition of *S. ecuadorensis* extract (Table 5), which is possibly a consequence of the decreased activity of the plant extract due to reaction with food components such as lipids, proteins, and carbohydrates [29,30]. After 10 days of storage, the number of these bacteria €n both samples was 3.3 log cfu/mL, being significantly lower than that reported for day 1, which could be due to the fact that despite its resistance to cleaning and disinfection procedures, it presents demanding growth requirements [23]. These bacteria had not been detected in C and E samples.

Microbial and chemical reactions that cause food spoilage are associated with changes in pH, making it a reliable indicator of food stability. After 10 days, the pH values of E, J, and EJ chicken burgers decreased (Table 6), probably due to the production of organic acids mainly by the lactic acid bacteria (LAB) [31,32]. However, the pH value of the control samples during storage increased significantly after 4 days. It can be explained by the degradation of proteins and amino acids and the formation of ammonia by the growth of some Gram-negative bacteria, which increases the pH [32,33].

### 3.3. Effect of Spray-Dried Extract on Microbial Growth in Chicken Broth during Storage

Assessment of antimicrobial potential of *S. ecuadorensis* spray-dried extract in inoculated chicken broth was developed. UHT chicken broth is a product that can be stored for several months without refrigeration, which is made possible by the combination of heat treatment with aseptic packaging. It was found that during all storage, AMB did not grow in C and J samples, but in E and EJ, the growth was significantly high (Table 7). In the chicken broth, the AMB count increased from 3.7 to 9 log cfu/g up to 14 days of storage; then, the number decreased significantly to 4.9 log cfu/g (29 days of storage), and finally, the increase was slower until the end of storage. This result could be due to the initial contamination of the extract.

Spray-dried extract demonstrated bactericidal effects against *C. jejuni*. On the first day of storage, the counts of this bacterium in the inoculated chicken broth were significantly lower in the sample to which the extract was added (EJ) (Table 8). From this day until the end of storage, the *C. jejuni* count was below the detection limit in sample with extract; however, in the J sample, it did not occur until day 52. It is observed that the extract is much more effective on *C. jejuni* in the broth than in the burgers, possibly due to the simplicity of the food matrix. Compounds present in the extract with potential antimicrobial activity can act more easily since they do not bind to food components such as lipids and proteins. *C. jejuni* did not grow in C and E samples (without inoculum), indicating that there was no presence of this microorganism.

### 3.4. Effect of Ethanol-Water Extract on Microbial Growth in Fish Burgers during Storage

Hake (*Merluccius merluccius*) is one of the most consumed fish species, with Spain being the largest market for it in the world [34]. In fish, due to the characteristics of its meat, a large group of bacteria can develop, including Gram-negative bacteria such as *Shewanella* [35,36], even more so if it is minced meat, which explains the high initial values of AMB in all the samples. Significant differences between the samples without (C) and with extract ETOH-H_2_O (E) were observed. The addition of 8% extract reduced the growth of AMB between days 7 and 9 of storage, reaching the same counts on day 14 as the samples without extract, around 8 log cfu/g. In addition, the growth of AMB in the inoculated samples (ES) was mainly reduced between days 3 and 9 of storage; in the samples with extract, the AMB count was approximately 3.5 log cycles lower, after which it began to grow and reached 7.9 log cfu/g at the end of refrigerated storage (Table 9).

The antimicrobial potential of *S. ecuadorensis* extract continued to be measured in fish burgers inoculated with *Shew. putrefaciens* (Table 10), which was chosen as specific microorganism for fish spoilage. Noninoculated samples (C and E) were not naturally contaminated with these bacteria. In the inoculated samples S and ES, the addition of extract had significant effect on the growth of this microorganism, showing an inhibitory effect throughout the storage time. A level of reduction in bacterial counts of up to 1 log was observed on day 1, and *Shew. putrefaciens* load was further reduced to approximately 2 logs on day 3, 4 logs on days 7 to 9, and 1 log at the end of the experiment (day 14).

Moreira et al. [28] indicated in their work on the *Simira* genus that there is still little published information on the phytochemical study of some species, but they found extracts of *Simira glaziovii* and *Simira sampaioana* with antimicrobial effect and MIC greater than 100 μg/mL against *Mycobacterium fortuitum*, *Mycobacterium tuberculosis*, and *Mycobacterium kansasii*. Wright et al. [37,38] mentioned that the methanolic extracts of *Terminalia ferdinandiana*, *Kunzea pomifera*, *Acronychia acidula*, *Citrus glauca*, and *Solanum aviculare* and the aqueous extracts of *K. pomifera*, *C. glauca*, and *Davidsonia pruriens* showed inhibition against *Shew. putrefaciens*. In addition, they mentioned that 0.5 mg/mL of the methanolic extract of *T. ferdinandiana* were effective in inhibiting total bacterial growth in a fish model system in a cold room. Other authors have also reported good inhibitory effects against *Shew. putrefaciens* from ethanolic extracts of *Urtica dioica* leaves in fish meat [19] or chitosan and gelatin-chitosan edible films that have incorporated clove essential oil in trout or dolphinfish fillets, respectively [33,39].

As shown in the Table 11, the initial pH value of the fish burgers corresponding to the control (C) was 6.69, similar to that reported in other studies [17,40], and is related to normal variability but higher than that reported by Schelegueda et al. [35] for Argentine hake burgers.

Fish burgers with inoculum (S) had a pH similar to the control sample (C), from day 1 to day 7 and increased significantly until the end of storage; this trend could be due to the accumulation of alkaline compounds, such as ammonia, trimethylamine, and other nitrogen-containing compounds that are formed from the breakdown of proteins and nucleotides in the muscle during the post-mortem period [17,41]. However, during 9 days of storage, the pH in the extract (E) and inoculated extract (ES) samples was significantly lower than in the control (C) and inoculated (S) samples, which can be attributed to the antimicrobial effect of the extract against microbial action and enzymatic deterioration [17,42,43].

### 3.5. Effect of Extract on Microbial Growth in Broth during Storage

Strains of *Shew. putrefaciens* are among those responsible for the deterioration of marine fish, it belongs to the group of psychotrophic, facultative anaerobic, Gram-negative bacteria [37,44]. It can grow under chilling conditions and produce hydrogen sulfide (H_2_S) and trimethylamine with the consequent discoloration, off-odors and flavors, slime formation, and changes in texture [19,36].

It can be observed that the extract effectively inhibited the growth of AMB (Table 12), with high reductions in counts (4–6 logarithmic cycles) of ES against S until day 131 of storage; moreover, the E and ES samples did not show significant differences between them. In sample C, it was possible to verify that cross-contamination was avoided. The inhibitory effect of ethanol-water extracts on the growth of *Shew. putrefaciens* is evident (Table 13); log/mL values were 7–8 times lower than in the control (S). Wright et al. [37] reported that compounds such as tannins, alkaloids, anthraquinones, flavonoids, polyphenolics, phytosterols, and saponins present in *Kunzea pomifera* extracts could be responsible for inhibiting the growth of *Shew. putrefaciens*. On the other hand, Moreira et al. [20] indicated that some substances isolated from the genus *Simira* are alkaloids, coumarins, steroids, iridoids, lignans, diterpenes, and triterpenes, some of which, as reported by Rondón et al. [15], could be associated with the antibacterial activity of an ethanolic extract of *S. ecuadorensis*. *Shew. putrefaciens* was not detected in fish broth samples C and E during the entire storage period.

## 4. Conclusions

Plants are an essential part of the life of ancestral peoples in Ecuador; they are used for medicinal, food, ritual, and other uses; therefore, it is important to generate scientific information about their properties. The evidence from this study suggests that *S. ecuadorensis* extracts showed antimicrobial activity against *Shew. putrefaciens*, *C. jejuni*, *L. mesenteroides*, *B. cereus*, *Y. enterocolitica*, and *C. perfringens*. Spray-dried extract had a high effectiveness against *C. jejuni* in chicken broth from day 1 but no effect against AMB in the same product. However, the extract had no effect against the same microorganism in chicken burgers. The ethanol-water extract was effective in fish products; in the case of burgers, it was possible to show the reduction of AMB growth between days 3 and 9 and a noticeable effect against *Shew. putrefaciens* until day 9 of storage. They also demonstrated a great antimicrobial activity against AMB and *Shew. putrefaciens* in fish broth for 131 days of storage. The evidence from this study suggests that *S. ecuadorensis* has potential as a novel food additive to increase the microbiological safety in chicken and fish foods.

## Figures and Tables

**Table 1:** Bacterial strains selected for this study and their culture conditions.

Microorganism	Bacterial Strain	Gram	Incubation Temperature (°C)	Broth	Test Agar
*Salmonella enterica subsp. Enterica*	HUBU-UBU 72732	-	37	TSB	Muller Hinton
*Escherichia coli*	CECT 501	-	37	BHI	Muller Hinton
*Shigella sonnei*	CECT 457	-	37	TSB	Muller Hinton
*Yersinia enterocolitica*	CECT 559	-	37	TSB	Muller Hinton
*Vibrio alginolyticus*	CECT 521	-	30	Nutrient broth + 2% NaCl	Nutrient agar + 2% NaCl
*Campylobacter jejuni*	CECT7572	-	42	BHI	Nutrient+ 0.5% blood
*Clostridium perfringens*	CECT 376	+	42	BHI	Nutrient + 0.5% blood
*Staphylococcus aureus*	CECT 30	+	37	BHI	Muller Hinton
*Listeria monocytogenes*	ILSI-4	+	37	BHI	Muller Hinton
*Listeria innocua*	CECT 910	+	37	BHI	Muller Hinton
*Bacillus cereus*	CECT 148	+	30	BHI	Muller Hinton
*Enterococcus faecalis*	CECT 481	+	37	BHI	Nutrient + 0.5% blood
*Brochothrix thermosphacta*	CECT 847	+	26	TSB + Lev0.6%	Muller Hinton
*Leuconostoc mesenteroides*	CECT 394	+	30	MRS	MRS
*Weissella viridescens*	CECT 283	+	30	MRS	MRS
*Pseudomonas putida*	CECT 3241	-	30	TSB	Muller Hinton
*Pseudomonas fluorescens*	CECT 378	-	30	TSB	Muller Hinton
*Aeromona caviae*	CECT 838	-	30	TSB	Muller Hinton
*Shewanella putrefaciens*	CECT 5346	-	30	TSB	Muller Hinton
*Shewanella* sp.	CECT 4640	-	30	TSB	Muller Hinton

CECT, Spanish Type Culture Collection; ILSI: International Life Science Institute; HUBU-UBU: Hospital of Burgos-University of Burgos.

**Table 2:** Antimicrobial activity of extracts of *S. ecuadorensis* expressed as the diameter of the inhibition area (mm).

Microorganism	Extracts ***				
	ETOH	H_2_O	ETOH-H_2_O	ATOM	Positive Control *
*Bacillus cereus*	11.0 **	ni	ni	ni	18.0
*Staphylococcus aureus*	ni	ni	ni	ni	15.0
*Escherichia coli*	ni	ni	ni	ni	15.0
*Listeria monocytogenes*	ni	ni	ni	ni	17.0
*Listeria innocua*	ni	ni	ni	ni	14.0
*Salmonella enterica*	ni	ni	ni	ni	14.0
*Shigella sonnei*	ni	ni	ni	ni	13.0
*Yersinia enterocolitica*	12.0	ni	ni	ni	16.0
*Brochothrix thermophacta*	ni	ni	ni	ni	17.0
*Pseudomonas putida*	ni	ni	ni	ni	19.0
*Pseudomonas fluorescens*	ni	ni	ni	ni	17.0
*Aeromona caviae*	ni	ni	ni	ni	19.0
*Shewanella putrefaciens*	8.0	12.0	12.0	8.0	36.0
*Shewanella* sp.	16.0	ni	ni	ni	29.0
*Clostridium perfringens*	12.0	ni	ni	ni	10.0
*Campylobacter jejuni*	10.0	18.0	10.0	10.0	17.0
*Vibrio alginolyticus*	ni	ni	ni	ni	14.0
*Enterococcus faecalis*	ni	ni	ni	ni	9.0
*Leuconostoc mesenteroides*	9.0	8.0	8.0	ni	19.0
*Weissella viridescens*	ni	ni	ni	ni	16.0

ni, no inhibition; * positive control: Sodium hypochlorite solution (0.94%); ** Average values of three replicates (*n* = 3); *** ETOH (extracted with ethanol), ETOH-H_2_O (extracted with ethanol-water), ATOM (extracted with water and spray-dried), and H_2_O (extracted with water and freeze- dried).

**Table 3:** Minimum inhibitory concentration (MIC) of extracts of *S. ecuadorensis* (mg/mL).

Extracts **				
Microorganism	ETOH	H_2_O	ETOH-H_2_O	ATOM
*Shewanella putrefaciens*	80 *	80	80	80
*Shewanella* sp.	40	_	_	_
*Campylobacter jejuni*	80	80	80	80
*Leuconostoc mesenteroides*	80	80	40	
*Yersinia enterocolitica*	20	_	_	_
*Bacillus cereus*	20	__	_	_
*Clostridium perfringens*	20	_	_	_

Concentration range 5–80 mg/mL; * Three replicates (*n* = 3); ** ETOH (extracted with ethanol), ETOH-H_2_O (extracted with ethanol-water), ATOM (extracted with water and spray-dried) and H_2_O (extracted with water and freeze-dried).

**Table 4:** Growth of AMB (log cfu/g) in chicken burgers for 10 days of storage: without inoculum€); with 8% spray-dried extr€ (E); with inoculum (J); with 8% of extract and inoculum (EJ).

Sample		C	E	J	EJ
Days	0	4.9 ± 0.07 D	---	---	---
	1	5.7 ± 0.10 bC	5.2 ± 0.08 cC	6.3 ± 0.04 AC	4.6 ± 0.21 dC
	4	8.2 ± 0.14 aB	7.1 ± 0.45 bB	8.5 ± 0.13 aB	6.3 ± 0.35 bB
	7	8.7 ± 0.11 aA	8.6 ± 0.26 abA	8.7 ± 0.12 abA	8.3 ± 0.19 bA
	10	8.9 ± 0.15 aA	8.8 ± 0.05 aA	8.8 ± 0.05 aA	8.6 ± 0.18 aA

Results are expressed as mean ± standard deviation (*n* = 3). a–d Different letters within the same row indicate statistical differences; A–D Different letters in the same column indicate significant differences along storage, *p* < 0.05.

**Table 5:** Growth of *C. jejuni* (log cfu/g) in chicken burgers for 10 days of storage: with inoculum (J); with 8% of spray-dried extract and inoculum (EJ).

Sample		J	EJ
Days	1	4.5 ± 0.22 aA	4.3 ± 0.30 aA
	4	4.6 ± 0.19 aA	4.5 ± 0.15 aA
	7	4.0 ± 0.09 aB	3.8 ± 0.51 aAB
	10	3.3 ± 0.18 aC	3.3 ± 0.20 aB

Results are expressed as mean ± standard deviation (*n* = 3). a Same letter within the same row indicate no statistical differences; A–C Different letters in the same column indicate significant differences along storage, *p* < 0.05.

**Table 6:** pH value of chicken burgers during storage for 10 days: without inoculum (C); with 8% spray-dried extract (E); with inoculum (J); and with 8% of spray-dried extract and inoculum (EJ).

Sample		C	E	J	EJ
Days	0	5.92 ± 0.01 B	---	---	---
	1	5.95 ± 0.02 abB	5.91 ± 0.02 bC	5.99 ± 0.01 aB	5.79 ± 0.01 cA
	4	6.33 ± 0.01 aA	6.32 ± 0.03 aA	5.78 ± 0.01 bC	5.75 ± 0.01 bA
	7	6.31 ± 0.01 aA	6.02 ± 0.13 bB	6.08 ± 0.01 bcA	4.97 ± 0.03 cB
	10	6.35 ± 0.02 aA	5.73 ± 0.01 cC	5.97 ± 0.01 bB	4.83 ± 0.03 dC

Results are expressed as mean ± standard deviation (*n* = 3). a–d Different letters within the same row indicate statistical differences; A–C Different letters in the same column indicate significant differences along storage, *p* < 0.05.

**Table 7:** Growth of AMB (log cfu/g) in chicken broth during storage for 85 days: with 8% spray-dried extract (E); with 8% of spray-dried extract and inoculum (EJ).

Sample		E	EJ
Days	0	---	---
	1	3.76 ± 0.25 aE	3.65 ± 0.14 aG
	4	4.68 ± 0.57 aE	5.43 ± 0.16 aEF
	7	8.02 ± 0.13 aAB	8.02 ± 0.13 aBC
	10	9.04 ± 0.04 aA	9.20 ± 0.09 aA
	14	9.12 ± 0.05 aA	8.89 ± 0.02 bAB
	29	4.86 ± 0.99 aDE	4.90 ± 0.32 aF
	52	6.30 ± 0.93 aCD	6.59 ± 0.19 aDE
	67	6.98 ± 0.15 aBC	6.42 ± 1.12 aDE
	85	6.71 ± 0.33 bBC	7.31 ± 0.23 aCD

Results are expressed as mean ± standard deviation (*n* = 3). a–b Different letters within the same row indicate statistical differences; A–F Different letters in the same column indicate significant differences along storage, *p* < 0.05.

**Table 8:** Growth of *C. jejuni* (log cfu/g) in chicken broth during storage for 85 days: with inoculum (J); with 8% of spray-dried extract and inoculum (EJ).

Sample		J	EJ
Days	1	3.65 ± 0.05 A	2.57 ± 0.05
	4	3.43 ± 0.47 AB	ND
	7	3.43 ± 0.47 AB	ND
	10	2.99 ± 0.02 B	ND
	14	2.79 ± 0.28 B	ND
	29	2.00 ± 0.31 C	ND
	52	ND	ND
	67	ND	ND
	85	ND	ND

Results are expressed as mean ± standard deviation (*n* = 3). A–C Different letters in the same column indicate significant differences along storage, *p* < 0.05. ND means below detection limit (<1 cfu/mL).

**Table 9:** Growth of AMB (log cfu/g) in fish burgers for 14 days of storage: without inoculum (C); with inoculum (S); with 8% ethanol-water extract (E); with 8% of extract and inoculum (ES).

Sample		C	E	S	ES
Days	0	4.1 ± 0.20 D	---	---	---
	1	3.9 ± 0.50 aD	4.1 ± 0.30 aC	4.4 ± 0.20 aD	3.8 ± 0.2 aD
	3	4.5 ± 0.10 bD	4.6 ± 0.12 bC	5.6 ± 0.12 aC	3.9 ± 0.15 cD
	7	5.9 ± 0.06 bC	4.4 ± 0.13 dC	8.3 ± 0.15 aB	4.9 ± 0.1 cC
	9	7.1 ± 0.10 bB	5.8 ± 0.16 cB	9.3 ± 0.13 aA	5.8 ± 0.21 cB
	14	8.2 ± 0.13 aA	7.8 ± 0.22 aA	8.3 ± 0.27 aB	7.9 ± 0.45 aA

Results are expressed as mean ± standard deviation (*n* = 3). a–d Different letters within the same row indicate statistical differences; A–D Different letters in the same column indicate significant differences along storage, *p* < 0.05.

**Table 10:** Growth of *Shew. putrefaciens* (log cfu/g) in fish burgers for 14 days of storage: with inoculum (S); with 8% of ethanol-water extract and inoculum (ES).

Sample		S	ES
Days	1	3.9 ± 0.08 aD	3.0 ± 0.30 bC
	3	5.2 ± 0.27 aC	3.1 ± 0.43 bC
	7	8.3 ± 0.06 aB	4.4 ± 0.23 bB
	9	9.2 ± 0.08 aA	5.3 ± 0.39 bB
	14	8.4 ± 0.10 aB	7.2 ± 0.45 bA

Results are expressed as mean ± standard deviation (*n* = 3). a–b Different letters within the same row indicate statistical differences; A–D Different letters in the same column indicate significant differences along storage, *p* < 0.05.

**Table 11:** pH in fish burgers for 14 days of storage: without inoculum (C); with 8% ethanol-water extract (E); with inoculum (S); and with 8% of extract and inoculum (ES).

Sample		C	E	S	ES
Days	0	6.69 ± 0.01 A	---	---	---
	1	6.55 ± 0.01 aBC	6.40 ± 0.00 bA	6.56 ± 0.03 aB	6.36 ± 0.06 bA
	3	6.57 ± 0.01 aB	6.35 ± 0.01 cAB	6.51 ± 0.01 bB	6.29 ± 0.00 dA
	7	6.57 ± 0.05 aB	6.36 ± 0.03 bAB	6.58 ± 0.04 aB	6.31 ± 0.01 bA
	9	6.45 ± 0.03 bCD	6.29 ± 0.03 cBC	6.92 ± 0.04 aA	6.28 ± 0.01 cA
	14	6.39 ± 0.01 bD	6.26 ± 0.00 bC	6.89 ± 0.08 aA	6.25 ± 0.01 bA

Results are expressed as mean ± standard deviation (*n* = 3). a–d Different letters within the same row indicate statistical differences; A–D Different letters in the same column indicate significant differences along storage, *p* < 0.05.

**Table 12:** Growth of AMB (log cfu/g) in fish broth during storage for 131 days: without inoculum (C); with 8% ethanol-water extract (E); with inoculum (S); and with 8% of extract and inoculum (ES).

Sample		C	E	S	ES
Days	0	ND	---	---	---
	1	ND	2.92 ± 0.10 bC	3.50 ± 0.21 aF	2.96 ± 0.05 bAB
	3	ND	2.94 ± 0.11 bC	6.92 ± 0.14 aE	2.55 ± 0.48 bB
	7	ND	2.94 ± 0.12 bC	8.98 ± 0.03 aBC	2.81 ± 0.29 bAB
	14	ND	2.79 ± 0.19 bC	8.79 ± 0.08 aBCD	2.84 ± 0.13 bAB
	21	ND	2.70 ± 0.34 bC	8.83 ± 0.24 aBCD	3.00 ± 0.08 bAB
	28	ND	2.68 ± 0.26 bC	8.50 ± 0.25 aCD	2.89 ± 0.13 bAB
	38	ND	2.85 ± 0.09 bC	9.06 ± 0.02 aB	2.91 ± 0.24 bB
	44	ND	2.90 ± 0.11 bC	8.97 ± 0.08 aBC	2.91 ± 0.40 bAB
	65	ND	3.55 ± 0.10 bB	9.30 ± 0.16 aAB	3.37 ± 0.26 bAB
	72	ND	3.12 ± 0.13 bBC	9.67 ± 0.25 aA	3.18 ± 0.10 bAB
	84	ND	2.77 ± 0.25 bC	8.83 ± 0.13 aBCD	3.08 ± 0.19 bAB
	104	ND	4.31 ± 0.38 bA	8.97 ± 0.34 aBC	3.75 ± 0.82 bA
	131	ND	2.85 ± 0.07 bC	8.33 ± 0.14 aD	2.94 ± 0.05 bAB

Results are expressed as mean ± standard deviation (*n* = 3). a–b Different letters within the same row indicate statistical differences; A–F Different letters in a column indicate significant differences between days of storage, *p* < 0.05. ND means below detection limit (<1 cfu/mL).

**Table 13:** Growth of *Shew. putrefaciens* (log cfu/g) in fish broth during storage for 131 days: with inoculum (S); with 8% of ethanol-water extract and inoculum (ES).

Sample		S	ES
Days	0	---	---
	1	3.42 ± 0.42 aF	2.21 ± 0.09 bA
	3	6.97 ± 0.06 aE	ND
	7	9.15 ± 0.15 aBC	2.08 ± 0.04 bA
	14	8.75 ± 0.08 aBCD	1.63 ± 0.15 bCD
	21	8.76 ± 0.05 aBCD	1.15 ± 0.15 bE
	28	8.40 ± 0.37 aCD	1.66 ± 0.18 bBCD
	38	8.81 ± 0.13 aBC	ND
	44	8.86 ± 0.04 aBC	2.24 ± 0.06 bA
	65	9.23 ± 0.09 aAB	1.90 ± 0.3 bABC
	72	9.72 ± 0.17 a	2.06 ± 0.1 bAB
	84	8.92 ± 0.12 aBCD	2.06 ± 0.15 bA
	104	8.56 ± 0.02 aBC	1.42 ± 0.10 bDE
	131	8.29 ± 0.10 aD	1.63 ± 0.2 bCD

Results are expressed as mean ± standard deviation (*n* = 3). a–b Different letters within the same row indicate statistical differences; A–F Different letters in the same column indicate significant differences along storage, *p* < 0.05. ND means below detection limit (<1 cfu/mL).

## Data Availability

Data is contained within the article.
